# Role of PCK2 in the proliferation of vascular smooth muscle cells in neointimal hyperplasia

**DOI:** 10.7150/ijbs.75577

**Published:** 2022-08-08

**Authors:** Dai Sik Ko, Junho Kang, Hye Jin Heo, Eun Kyoung Kim, Kihun Kim, Jin Mo Kang, YunJae Jung, Seung Eun Baek, Yun Hak Kim

**Affiliations:** 1 Division of Vascular Surgery, Department of General Surgery, Gachon University Gil Medical Center, Incheon, Republic of Korea.; 2 Medical Research Institute, Pusan National University, Busan, Republic of Korea.; 3 Department of Anatomy, School of Medicine, Pusan National University, Yangsan, Republic of Korea.; 4 Department of Occupational and Environmental Medicine, Kosin University Gospel Hospital, Republic of Korea.; 5 Department of Microbiology, College of Medicine, Gachon University, Incheon, Republic of Korea.; 6 Lee Gil Ya Cancer and Diabetes Institute, Gachon University, Incheon, Republic of Korea.; 7 Department of Health Science and Technology, Gachon Advanced Institute for Health Science & Technology, Gachon University, Incheon, Republic of Korea.; 8 Department of Biomedical Informatics, School of Medicine, Pusan National University, Yangsan, Republic of Korea.

**Keywords:** vascular smooth muscle cell proliferation, neointimal hyperplasia, atherosclerosis, PCK2, Akt-FoxO-PCK2 pathway

## Abstract

Vascular smooth muscle cell (VSMC) proliferation is a hallmark of neointimal hyperplasia (NIH) in atherosclerosis and restenosis post-balloon angioplasty and stent insertion. Although numerous cytotoxic and cytostatic therapeutics have been developed to reduce NIH, it is improbable that a multifactorial disease can be successfully treated by focusing on a preconceived hypothesis. We, therefore, aimed to identify key molecules involved in NIH via a hypothesis-free approach. We analyzed four datasets (GSE28829, GSE43292, GSE100927, and GSE120521), evaluated differentially expressed genes (DEGs) in wire-injured femoral arteries of mice, and determined their association with VSMC proliferation *in vitro*. Moreover, we performed RNA sequencing on platelet-derived growth factor (PDGF)-stimulated human VSMCs (hVSMCs) post-phosphoenolpyruvate carboxykinase 2 (*PCK2*) knockdown and investigated pathways associated with PCK2. Finally, we assessed NIH formation in *Pck2* knockout (KO) mice by wire injury and identified *PCK2* expression in human femoral artery atheroma. Among six DEGs, only *PCK2* and *RGS1* showed identical expression patterns between wire-injured femoral arteries of mice and gene expression datasets. PDGF-induced VSMC proliferation was attenuated when hVSMCs were transfected with* PCK2* siRNA. RNA sequencing of *PCK2* siRNA-treated hVSMCs revealed the involvement of the Akt-FoxO-PCK2 pathway in VSMC proliferation via Akt2, Akt3, FoxO1, and FoxO3. Additionally, NIH was attenuated in the wire-injured femoral artery of *Pck2*-KO mice and *PCK2* was expressed in human femoral atheroma. PCK2 regulates VSMC proliferation in response to vascular injury via the Akt-FoxO-PCK2 pathway. Targeting PCK2, a downstream signaling mediator of VSMC proliferation, may be a novel therapeutic approach to modulate VSMC proliferation in atherosclerosis.

## Introduction

Atherosclerosis is the leading cause of some of the deadliest diseases, such as myocardial infarction and stroke, worldwide 1. Pathologically, it involves multiple processes, such as oxidative stress, endothelial dysfunction, lipid infiltration, chronic inflammation, cell proliferation and migration 2. Several studies have suggested that aberrant vascular smooth muscle cells (VSMCs) proliferation and neointima formation dominate the development of atherosclerotic lesions 3. Blood vessel walls are majorly composed of VSMCs, which are crucial in maintaining the normal physiological function of blood vessels 4. Notably, phenotype switching from contractile VSMCs to synthetic VSMCs increases the proliferation and migration of VSMCs and the expression of the extracellular matrix. Moreover, this is an major step in the growth of intimal hyperplasia 5, 6.

Understanding the molecular and cellular mechanisms underlying VSMC proliferation in atherosclerosis is a cornerstone for the development of antiproliferative therapeutic approaches. Studies have investigated various cell cycle-related proteins and pathways in association with VSMC proliferation. For example, overexpression of p21, a cyclin-dependent kinase inhibitor 1, using an adenoviral vector in balloon-injured porcine arteries limited the development of intimal hyperplasia 7. Moreover, inhibition of cyclin-dependent kinase 2 and repression of cyclin A gene transcription, via the induction of p27, inhibited VSMC proliferation in rat carotid artery after balloon angioplasty 8. Moreover, mounting evidence suggests that noncoding RNA plays critical roles in regulating VSMC proliferation. For instance, long intergenic noncoding RNA-p21 represses VSMC proliferation and induces cell apoptosis by enhancing p53 activity 9. Additionally, miR-378a-5p promoted VSMC proliferation by repressing the *CDK1* gene expression 10.

In the clinical scenario, two drugs are widely used to treat atherosclerosis. They are delivered directly to the blood vessel wall in the form of coated balloon catheters and stents. One of these drugs, rapamycin, is a macrocyclic triene antibiotic that is the product of a natural fermentation process. Rapamycin interferes with mTOR and its downstream signaling cascades, reducing protein synthesis and causing concomitant cell cycle arrest 11. In several clinical trials, implantation of rapamycin-coated stents on the coronary artery caused a considerable decrease in the restenosis rate and cardiovascular events 12. The second drug, paclitaxel, is a potent antimicrotubule agent that promotes the development of stable dysfunctional microtubules that disrupt cellular processes 13. Paclitaxel-coated balloons and stents are superior in targeting lesion revascularization to non-paclitaxel-coated devices and, therefore, have been widely accepted in clinical practice 14. These advances in target lesion revascularization in peripheral arterial disease have decreased major adverse limb events, such as amputation and development of acute limb ischemia or critical limb ischemia, post-revascularization. However, there are still unmet needs, such as in-stent restenosis, and repetitive interventions are required to maintain patency 15. It is crucial to emphasize that it is improbable that a multifactorial disease, such as atherosclerosis, can be successfully treated by focusing on a preconceived hypothesis of cytotoxic or cytostatic mechanisms. Therefore, in this study, we use a hypothesis-free approach to identify a key regulator of VSMC proliferation in response to vascular injury, analyze its pathway, and verify its function *in vitro* and *in vivo*.

## Materials and Methods

### Data acquisition

We obtained the gene expression profiles for carotid atheroma from the Gene Expression Omnibus database (GEO; http://www.ncbi.nlm.nih.gov/geo/). The accession numbers were GSE43292^16^, GSE28829 17, GSE100927 18, and GSE120521 19. Although the GSE120521 dataset included gene expression profiles obtained by high throughput sequencing, the remaining three datasets included gene expression profiles obtained by array sequencing. Two of these datasets, GSE43292 and GSE100927, contained gene expression data from disease (carotid atheroma or atherosclerotic carotid artery) groups and/or normal (intact carotid artery) groups. Additionally, GSE28829 included gene expression data associated with early and advanced carotid atheroma plaques, whereas GSE120521 included gene expression data associated with stable and unstable carotid atheroma plaques. Detailed characteristics of these datasets are presented in Supplementary Table 1.

### Data preprocessing

There is an inevitable heterogeneity in the data published in the GEO database. We performed data preprocessing using the “rma,” “voom,” and “loess normalization” methods provided by the “limma” package of R software (v 3.42.2) to minimize heterogeneity 20. We converted the probe ID into a gene symbol and used it, and when several probes were matched with one gene symbol, the average level of the probe was used as the expression value of the corresponding gene. In addition, the absence of a gene symbol was excluded from this study.

### Identification of differentially expressed genes

To identify the differentially expressed genes (DEGs) between carotid atheroma and normal tissues in the GEO dataset, we conducted linear regression analysis using the “limma” package. We considered genes with an adjusted *P* value < 0.05 to have a significant differential expression between the two groups. The false discovery rate (FDR) method was used to calculate the adjusted *P* value. The log_2_ fold change > 0 was the threshold for identifying up-regulated genes, and a log_2_ fold change < 0 was the threshold for identifying down-regulated genes. To screen for the intersectional genes significantly expressed in each cohort, we used the R package “venn” (v1.10) to plot Venn diagrams.

### Identification of potential *PCK2*-related genes and signaling pathways

We performed DEG analysis using RNA-seq data from cells harboring phosphoenolpyruvate carboxykinase 2 (*PCK2*) knockdown to identify genes potentially associated with *PCK2*. We performed DEG analysis using the “limma” package, and the criteria for *PCK2* associated DEG were defined as adjusted *P <* 0.05 and -2 > log_2_ fold change > 2. To identify the potential signaling pathways associated with PCK2, we performed a Kyoto Encyclopedia of Genes and Genomes (KEGG) pathway enrichment analysis using the R package "clusterProfiler" (v 3.14.3) 21. The cut-off values for KEGG analysis were set according to parameters, and terms with ≥ 5 associated genes and having an adjusted p-value < 0.05 were included for subsequent analyses.

### Animal experimentation and ethics statement

All experiments involving animals complied with the guidelines provided by the Guide for the Care and Use of Laboratory Animals published by the US National Institute of Health (NIH Publication No. 85-23, 2011 revision). All animal experiment protocols were permissioned by the Pusan National University Institutional Animal Care and Use Committee of the College of Medicine (PNU-2020-2680). We performed genotyping, including that of *Pck2*-deficient mice, by PCR, using the protocol provided by the Jackson Laboratory (Harlan Nossan, ITA). We purchased C57BL/6J wild-type control mice (WT) from the Jackson Laboratory. All animals were housed in an air-conditioned room at 22-25 °C and maintained under a 12 h light/dark cycle. Food and water were provided *ad libitum.*

All human experiments in this study complied with the Declaration of Helsinki and were approved by the locally appointed ethics committee of the Gachon University Gil Medical Center (GFIRB2020-085). Informed consent was obtained from all the study participants.

### Vascular injury models and blood flow measurement

We anesthetized the WT and *Pck2*-deficient male mice (seven weeks old) via an intraperitoneal injection of chloral hydrate (450 mg/kg). Efficiency of an anesthetic dose was confirmed by the response to a toe pinch. Subsequently, we injured them in the right femoral artery using a 0.26 mm diameter angioplasty guidewire with hydrophilic coating (ASAHI RG3, Asahi Intecc, Japan) under aseptic conditions, as previously described 22. The wire-injured femoral arteries were harvested from mice euthanized by CO_2_ insufflation four weeks after the injury. Isolated femoral arteries were fixed overnight in 4% paraformaldehyde at 4 °C, and their paraffin-embedded cross sections (4 μm) were prepared. These tissue sections were stained with hematoxylin and eosin (H&E) and specific antibodies for immunohistological analysis. The neointimal volume was measured using an Image J, version 1.53m, program (NIH Image, Bethesda, MD). Blood flow in the femoral artery was measured using a laser Doppler perfusion imaging (LDPI) analyzer (Moor Instruments, Devon, UK) at 0 (before wire-injury), 1, 2, 3, and 4 weeks after the femoral artery injury. Blood flow was quantified using pixel values of LDPI color.

### Chemical reagents and antibodies

Antibodies against PCK2 (Cat #. NBP2-75610), KIAA1671 (Cat #. NBP1-91085), FHL1 (Cat #. NB100-1460), and LAMA5 (Cat #. NB300-144) were purchased from Novus Biologicals (Littleton, CO, USA), and those against RGS1 (Cat #. MBS851803) and AMPD3 (Cat #. MBS766604) were purchased from MyBioSource (San Diego, CA, USA). Platelet-derived growth factor (PDGF) and α-smooth muscle actin (α-SMA) (Cat #. A2547) were purchased from Millipore-Sigma (St. Louis, MO, USA). Antibodies against Ki67 (Cat #. SC-7846), PCNA (Cat #. SC-56), and β-actin (Cat #. SC-47778) were purchased from Santa Cruz Biotechnology, Inc. (Beverly, MA, USA). The PI3K inhibitor (LY294002; LY) and the Akt inhibitor (Akt inhibitor IV; AI) were purchased from Calbiochem (La Jolla, CA, USA). Antibodies against Akt1 (Cat #. 75692S), Akt2 (Cat #. 5239S), Akt3 (Cat #. 14982S), FoxO1 (Cat #. 2880S), FoxO3 (Cat #. 12829S), FoxO4 (Cat #. 9472S), and MitoTracker (Cat #. 8778S) were purchased from Cell Signaling Technology (Beverly, MA, USA). Phospho-FoxO1 (Cat #. ab131339), phospho-FoxO3 (Cat #. ab47285), phospho-FoxO4 (Cat #. ab47278), COXIV (Cat #. ab33985), and β-tubulin (Cat #. ab131205) antibodies were purchased from Abcam (Cambridge, UK). The PCK2 inhibitor PEPCKi was obtained from Axon Medchem (Reston, VA, USA). Horseradish peroxidase (HRP)-conjugated IgG antibody (Santa Cruz Biotechnology, Inc.) was used as the secondary antibody.

### Cell culture

Human aortic vascular smooth muscle cells (hVSMCs) were obtained from the American Tissue Culture Collection (Manassas, VA, USA). We cultured the hVSMCs in culture dishes containing a Medium-231 (M231500, Gibco BRL, Grand Island, NY, USA), 10% fetal bovine serum, 1% antibiotic-antimycotic solution (Gibco BRL) and smooth muscle growth supplement (S00725, Gibco BRL). Cells were incubated at 37 °C in a humidified atmosphere containing 5% CO_2_.

### Western blot analysis

We prepared hVSMC lysates in an ice-cold RIPA lysis buffer (Thermo Fisher Scientific, Rockford, IL, USA). The cytosolic and mitochondrial proteins of hVSMCs were extracted with Mitochondria/Cytosol Fractionation Kit (Cat #. ab65320, Abcam) according to the manufacturer's protocol. Subsequently, equal amounts (30 μg) of protein were separated by 8-10% SDS-PAGE under reducing conditions and then transferred to nitrocellulose membranes (Amersham Pharmacia Biotech, Piscataway, NJ, USA). The protein transferred membrane was blocked with 5% skimmed milk in Tris-buffered saline-Tween-20 (TBST) for 2 h at room temperature. Thereafter, they were incubated overnight with primary antibodies (1:1000) diluted in 5% skim milk at 4 °C. The following day, the blots were washed with TBST and were incubated with HRP-conjugated secondary antibody (1:3000) for 2 h at room temperature. Subsequently, blots were developed using the chemiluminescent ECL detection reagent (Thermo Fisher Scientific). An anti-β-actin antibody was used as an internal control. Protein bands were quantified using the UN-SCAN-IT GEL 7.1 program (Silk Scientific, UT, USA), and protein expression levels were calculated in relation to the β-actin protein expression.

### Reverse transcription (RT)-PCR analysis

Total RNA was isolated from the cells using the TRIzol reagent (Invitrogen, Carlsbad, NY, USA), according to the manufacturer's protocol. The isolated RNA (1 μg) by applying the ImProm-II reverse transcription system (Promega, Madison, WI, USA) was reverse transcribed into cDNA. Subsequently, cDNA amplification was performed using *PCK2*-specific primers (forward, 5′-GGG TGC TAG ACT GGA TCT GC-3′ and reverse, 5′-CTG GTT GAC CTG CTC TGT CA-3′). Equal amounts (5 μL) of PCR products were identified on a 0.8% agarose gel containing ethidium bromide. PCR products were quantified using the UN-SCAN-IT GEL 7.1 program, and the mRNA expression *of PKC2* was calculated in relation to that of *GAPDH*.

### Small interfering RNA (siRNA) preparation and transfection

We designed and synthesized siRNAs for *PCK2*,* RGS1*,* Akt1*,* Akt2*,* Akt3*,* FoxO1,* and *FoxO3* and scrambled siRNA as a negative control (Cat no. SN-1003) using an AccuTarget siRNA construction kit (Bioneer, Daejeon, Korea). All siRNAs were transfected through the pores of the cell membrane using Lipofectamine 2000 (Invitrogen) according to the manufacturer's protocol.

### RNA extraction, library construction, and sequencing

Total RNA (0.5 μg) was extracted from the transfected hVSMCs using the TRIzol reagent kit (Invitrogen), and the integrity of the RNA was assessed using the TapStation RNA screen tape. And then, RNA libraries were prepared using the Illumina TruSeq Stranded Total RNA Library Prep Gold Kit (Illumina, Inc., San Diego, CA, USA). The cleaved RNA fragment was copied into first-strand cDNA using random primers (Invitrogen) and reverse transcriptase (Invitrogen, Carlsbad, CA, USA). Finally, the cDNA library that passed the quality check was sequenced using the Illumina NovaSeq platform (Illumina, Inc., San Diego, CA, USA). All kits were used according to the manufacturer's protocol.

### RNA-seq analysis

We preprocessed the raw reads obtained from the sequencer to remove low quality reads and adapter sequences before performing the RNA-seq analysis. We aligned the processed reads to *Homo sapiens* (hg38) genome sequence using HISAT v2.1.0(1). This reference genome and the relevant annotation data were downloaded from the University of California Santa Cruz table browser (http://genome.uscs.edu). After alignment, StringTie v1.3.4 was used to estimate transcript abundance in each sample. The estimated transcript abundance table was used for subsequent analysis.

### MTT assay

Proliferation rates of hVSMCs were analyzed using the MTT assay. Briefly, 1 × 10^5^ cells were cultured in MTT solution (EZ-Cytox, Daeil Laboratories, Seoul, Republic of Korea)-treated medium according to the manufacturer's protocol. Cells were incubated at 37 °C in a humidified atmosphere containing 5% CO_2_. Optical density of the formazan crystals was measured at 450 nm using a microplate reader (TECAN SUNRISE, Mannedorf, Switzerland). Cells were counted using a hemocytometer. Relative proliferation rates were analyzed by comparing PDGF-treated cells with control cells.

### Immunofluorescence analysis

We incubated serial paraffin-sectioned (4 μm) femoral arteries with mouse anti-α-SMA (1:400) and either rabbit-anti PCK2 (1:200), rabbit-anti RGS1 (1:200), rabbit-anti AMPD3 (1:200), rabbit-anti KIAA1671 (1:200), goat-anti FHL1 (1:200), or rabbit-anti LAMA5 (1:200) antibodies. hVSMCs, plated on glass coverslips, were fixed with 4% paraformaldehyde, and nonspecific binding sites were blocked with 1% bovine serum albumin. The fixed cells were then incubated with MitoTracker, rabbit anti-PCK2 (1:400), and mouse anti-β-tubulin (1:400) antibodies. Goat anti-mouse Alexa488-conjugated IgG and Goat anti-mouse Alexa594-conjugated IgG or Donkey anti-goat Alexa 594-conjugated IgG (Abcam) were used to label the immunofluorescence signals for α-SMA, PCK2, RGS1, AMPD3, KIAA1671, FHL1, and LAMA5, respectively. After nuclei were stained with DAPI at 0.1 μg/mL, slides were mounted on a Vectashield, and fluorescence images were visualized using a scanning confocal microscope (LSM 510, Carl Zeiss, Oberkochen, Germany).

### Human femoral atheroma sampling

Five patients who underwent common femoral endarterectomy at Gachon University Gil Medical Center participated in this study. The femoral endarterectomy samples were collected from the participants in the surgery room and immediately frozen in liquid nitrogen for histological examination. Tissue fixation, H&E staining, and immunofluorescence staining were performed as mentioned in the previous sections.

### Statistical analysis

Results are expressed as the mean ± SEM. One-way analysis of variance (ANOVA) followed by Dunnett's multiple comparison test was used to determine the significance of the experimental results. Obtained data were analyzed using GraphPad Prism version 5.5.01. (GraphPad Software, USA). Statistical significance was set at *P <* 0.05.

## Results

### Common DEGs for each GEO dataset

**Figure 1:**
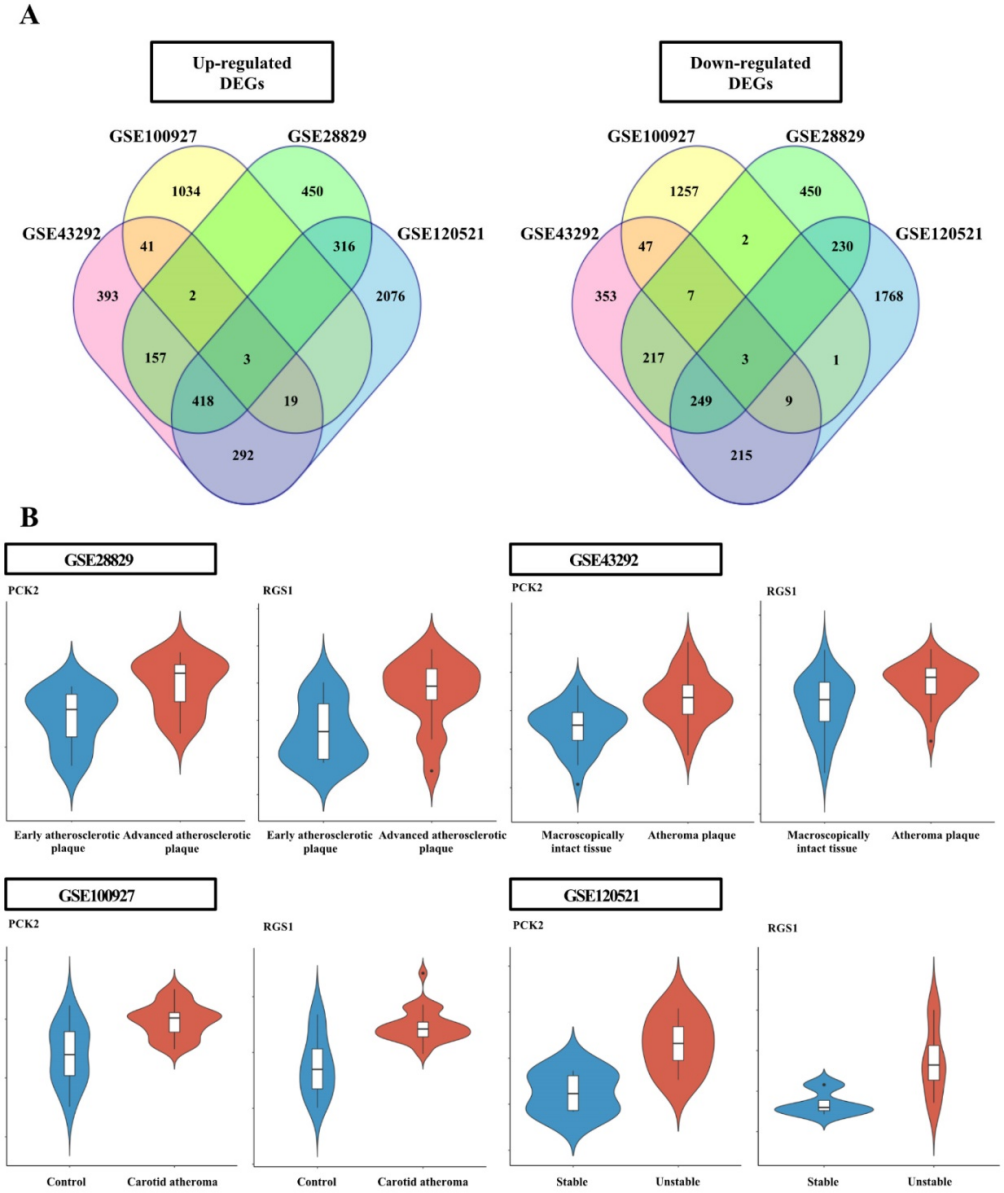
** Analysis of differentially expressed genes (DEGs) in each dataset. (A)** Venn diagram of the DEGs identified in each Gene Expression Omnibus dataset. The four datasets shared six DEGs, including three upregulated and three downregulated genes. **(B)** Violin plots from each dataset for PCK2 and RGS1 validated in wire-injured femoral arteries. Violin plots indicating PCK2 and RGS1 expression levels in atheromatous plaques and macroscopically intact tissues.

The DEG analysis data for individual datasets are provided in Supplementary Table 2-5. Six genes, common among the individual dataset, were identified (Figure 1); the three upregulated genes were adenosine monophosphate deaminse3 (*AMPD3*),* PCK2*, and regulator of G protein signaling 1 (*RGS1*); the three down-regulated genes were four and a half LIM domains 1 (*FHL1*), luminin subunit alpha 5 (*LAMA5*), and* KIAA1671* (Figure S1).

### Expression of *PCK2* and *RGS1* is associated with neointima hyperplasia in wire-injured vasculature

**Figure 2:**
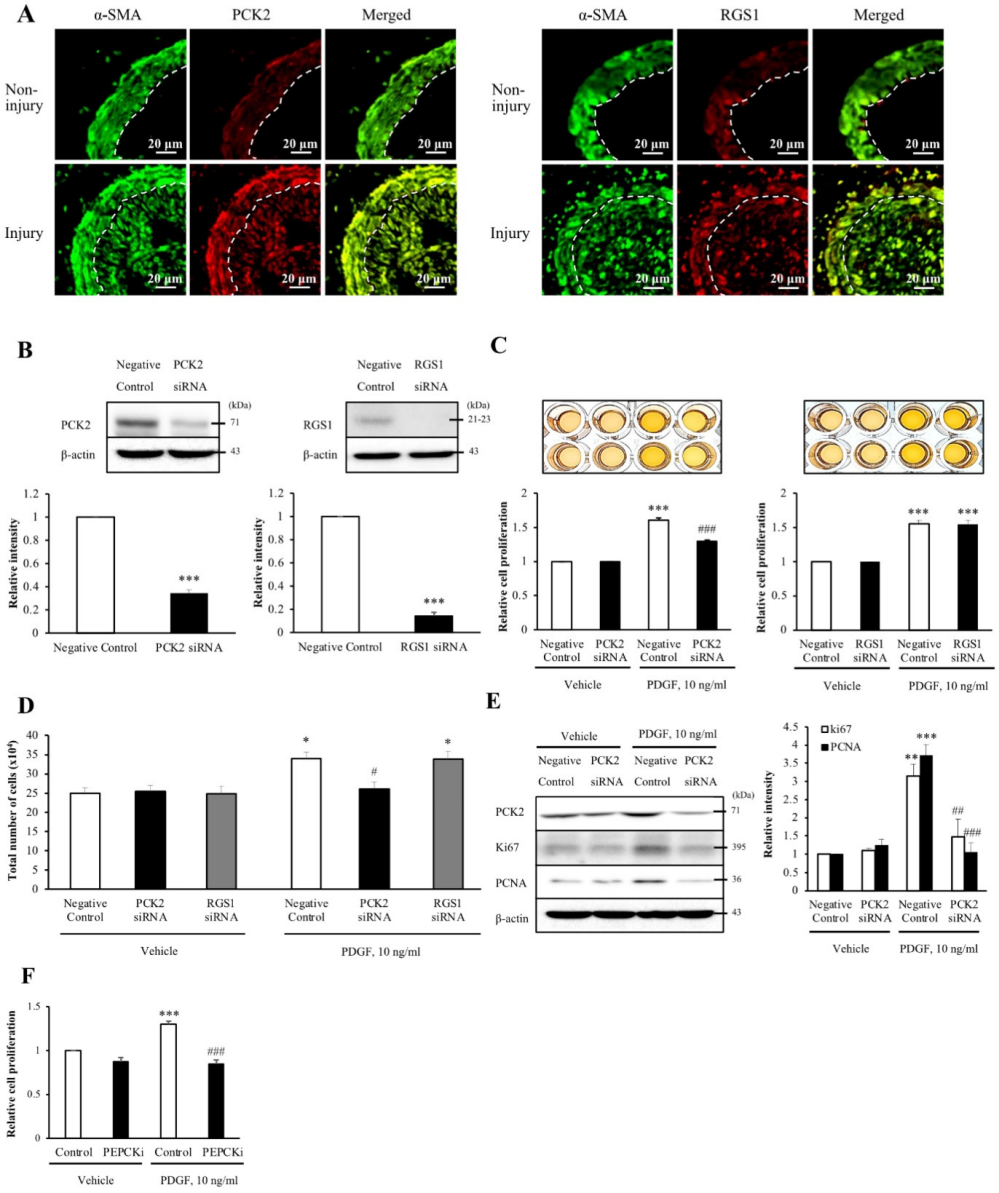
** Involvement of PCK2 and RGS1 in vasculature injury. (A)** PCK2 and RGS1 in the non-injury and injury media and neointima were stained with anti-PCK2 antibody and anti-RGS1 antibody, respectively. The protein levels of non-injured and injured media were compared. Vascular smooth muscle cells (VSMCs) were stained with anti-α-SMA antibody. Dashed line defines the media and neointima layer. Images are representative of 5-6 independent experiments. **(B)** VSMCs were transfected with negative control, PCK2, or RGS1 siRNA for 48 h. The protein levels of PCK2 and RGS1 were determined by western blotting using β-actin as an internal control. Blots are representative of four independent experiments. Relative band intensities are expressed as the means ± SEMs. ***P < 0.001 vs. corresponding value in negative control. **(C and D)** VSMCs were transfected with negative control, PCK2, and RGS1 siRNA for 48 h. These cells were treated with PDGF (10 ng/ml) for 24 h, and cell proliferation was determined using the MTT assay. Relative cell proliferation determined using the MTT and cell counting assays was expressed as means ± SEMs of four independent experiments. *P < 0.05 and ***P < 0.001 vs. corresponding value in PDGF-untreated negative control, #*P <* 0.05 and ###*P <* 0.001 vs. corresponding value in PDGF-treated negative control. **(E)** Protein expression of cell cycle-related antigens, PCNA and Ki67, was measured by western blotting. VSMCs were transfected with negative control or PCK2 siRNA for 48 h and stimulated with PDGF (10 ng/ml) for 24 h. The protein levels of PCK2, PCNA, and Ki67 were determined using β-actin as an internal control. Blots are representative of three independent experiments. Relative intensities were expressed as the means ± SEMs. **P < 0.01 and ***P < 0.001 vs. corresponding value in PDGF-untreated negative control, ##*P <* 0.01 and ###*P <* 0.001 vs. corresponding value in PDGF-treated negative control. **(F)** VSMCs were pre-treated with PEPCKi (10 µM) for 24 h and subsequently with PDGF (10 ng/ml) for 24 h. Cell proliferation was estimated by the MTT assay. Relative cell proliferation is expressed as the means ± SEMs of six independent experiments. ***P < 0.001 vs. corresponding value in PDGF-untreated control, ###*P <* 0.001 vs. corresponding value in PDGF-treated control

To assess whether the six DEGs were associated with neointimal hyperplasia, we evaluated the wire injuries in the C57BL/6J mice. At 4 weeks post-wire injury, the endothelium of the femoral artery was denuded and proliferation and migration of VSMCs resulted in extensive neointimal hyperplasia. To identify the cell types that expressed the DEGs in the blood vessel, we performed co-localization studies of α-SMA, a VSMC marker. Additionally, the expression of DEGs in non-injured media and injured media was compared by immunofluorescence (Figure 2A and Figure S2). Of the three up-regulated DEGs, the expression levels of *PCK2* and *RGS1* were higher in the injured media than that in the non-injured media. Contrarily, expression levels of *AMPD3* were lower in the injured media than in the non-injured media. However, all the downregulated DEGs showed higher expression in the injured media than in the non-injured media. Both *PCK2* and *RGS1* colocalized with α-SMA in neointimal hyperplasia (Figure 2A).

### Inhibition of *PCK2* attenuates PDGF-induced VSMC proliferation

Upon investigating the role of *PCK2* and *RGS1* in VSMC proliferation, PCK2 and RGS1 protein expression was attenuated in siRNA-transfected human VSMCs (hVSMCs) (siRNA concentration, 200 nM, 48 h; Figure 2B). MTT assay was performed to investigate PDGF (10 ng/mL for 24 h) induced VSMC proliferation. Notably, PDGF-induced increase in VSMC proliferation was considerably attenuated by *PCK2* siRNA, but not by *RGS1* siRNA in MTT assay (Figure 2C) and was evident from cell counting (Figure 2D). PDGF-induced expression of the cell cycle-related antigens, PCNA and Ki67, was also substantially attenuated in hVSMCs transfected with *PCK2* siRNA (Figure 2E). The PCK2 inhibitor, PEPCKi (10 μM), attenuated PDGF-induced proliferation of hVSMCs (Figure 2F).

### *PCK2*-related genes and signaling pathways

Using DEG analysis, we identified 314 genes potentially associated with *PCK2*, of which, 167 were upregulated and 147 were downregulated (Table S6 and S7). We identified five pathways that were considerably enriched with DEGs in the KEGG pathway enrichment analysis (Table S8). These pathways included the PI3K-Akt signaling pathway, FoxO signaling pathway, calcium signaling pathway, IL-17 signaling pathway, and microRNAs in cancer. The detailed characteristics of these signaling pathways are listed in Table S8. *PCK2* was involved in both PI3K-Akt and FoxO signaling pathways.

### The Akt-FoxO-PCK2 signaling pathway in PDGF-induced VSMC proliferation

**Figure 3:**
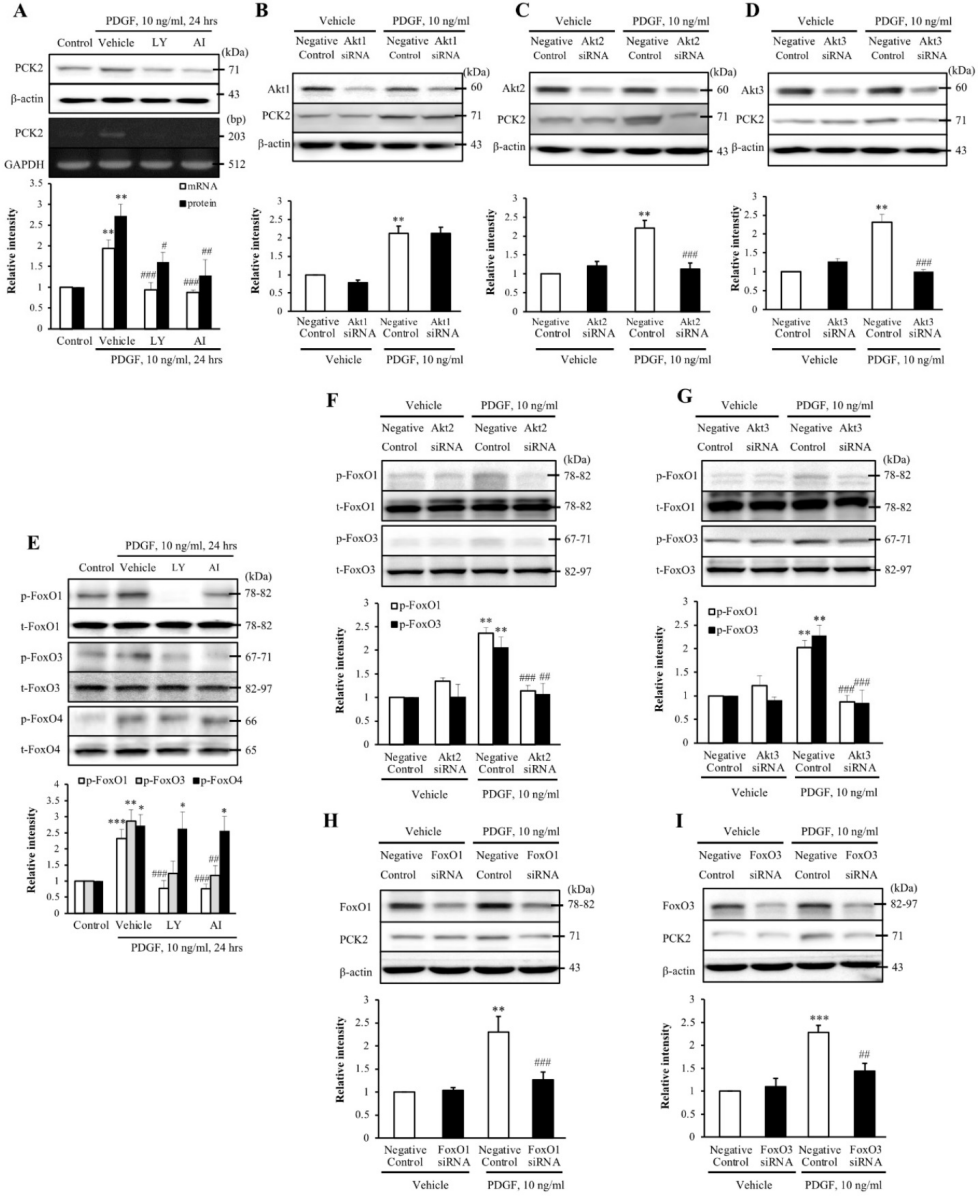
** Identification of the role played by PCK2 in PDGF-induced proliferation of vascular smooth muscle cells. (A)** Vascular smooth muscle cells (VSMCs) were pre-treated with LY (10 µM) or AI (10 µM) for 1 h, and the protein and mRNA levels of PCK2 were determined by western blotting and RT-PCR, respectively. β-actin and GAPDH were used as internal controls. Blots are representative of five independent experiments. Relative intensities are expressed as the mean ± SEMs. **P < 0.01 vs. corresponding value in control, #*P <* 0.05, ##*P <* 0.01, and ###*P <* 0.001 vs. corresponding value in vehicle. **(B-D)** VSMCs were transfected with negative control, Akt1, Akt2, or Akt3 siRNA for 48 h, and then stimulated with PDGF (10 ng/ml) for 24 h. The protein levels of PCK2 were determined by western blotting using β-actin as an internal control. Blots are representative of three independent experiments. Relative intensities are expressed as the means ± SEMs. **P < 0.01 vs. corresponding value in PDGF-untreated negative control, ###*P <* 0.001 vs. corresponding value in PDGF-treated negative control. **(E)** VSMCs were pre-treated with LY (10 µM) or AI (10 µM) for 1 h, and then the protein levels of phospho-FoxO1, phospho-FoxO3, and phospho-FoxO4 were determined by western blotting. Total-FoxO1, total-FoxO3, and total-FoxO4 were used as internal controls. Blots are representative of six independent experiments. Relative intensities are expressed as the mean ± SEMs. *P < 0.05, **P < 0.01, and ***P < 0.001 vs. corresponding value in control, ##*P <* 0.01 and ###*P <* 0.001 vs. corresponding value in vehicle. **(F and G)** VSMCs were transfected with negative control, Akt2, or Akt3 siRNA for 48 h and then stimulated with PDGF (10 ng/ml) for 24 h. The protein levels of phospho-FoxO1 and phospho-FoxO3 were determined by western blotting using total-FoxO1 and total-FoxO3 as internal controls. Blots are representative of six independent experiments. Relative intensities are expressed as the means ± SEMs. **P < 0.01 vs. corresponding value in PDGF-untreated negative control, ##*P <* 0.01 and ###*P <* 0.001 vs. corresponding value in PDGF-treated negative control. **(H and I)** VSMCs were transfected with negative control, FoxO1 or FoxO3 siRNA for 48 h, and then stimulated with PDGF (10 ng/ml) for 24 h. The protein levels of PCK2 were determined by western blotting using β-actin as an internal control. Blots are representative of six independent experiments. Relative intensities are expressed as the means ± SEMs. **P < 0.01 and ***P < 0.001 vs. corresponding value in PDGF-untreated negative control, ##*P <* 0.01 and ###*P <* 0.001 vs. corresponding value in PDGF-treated negative control.

**Figure 4:**
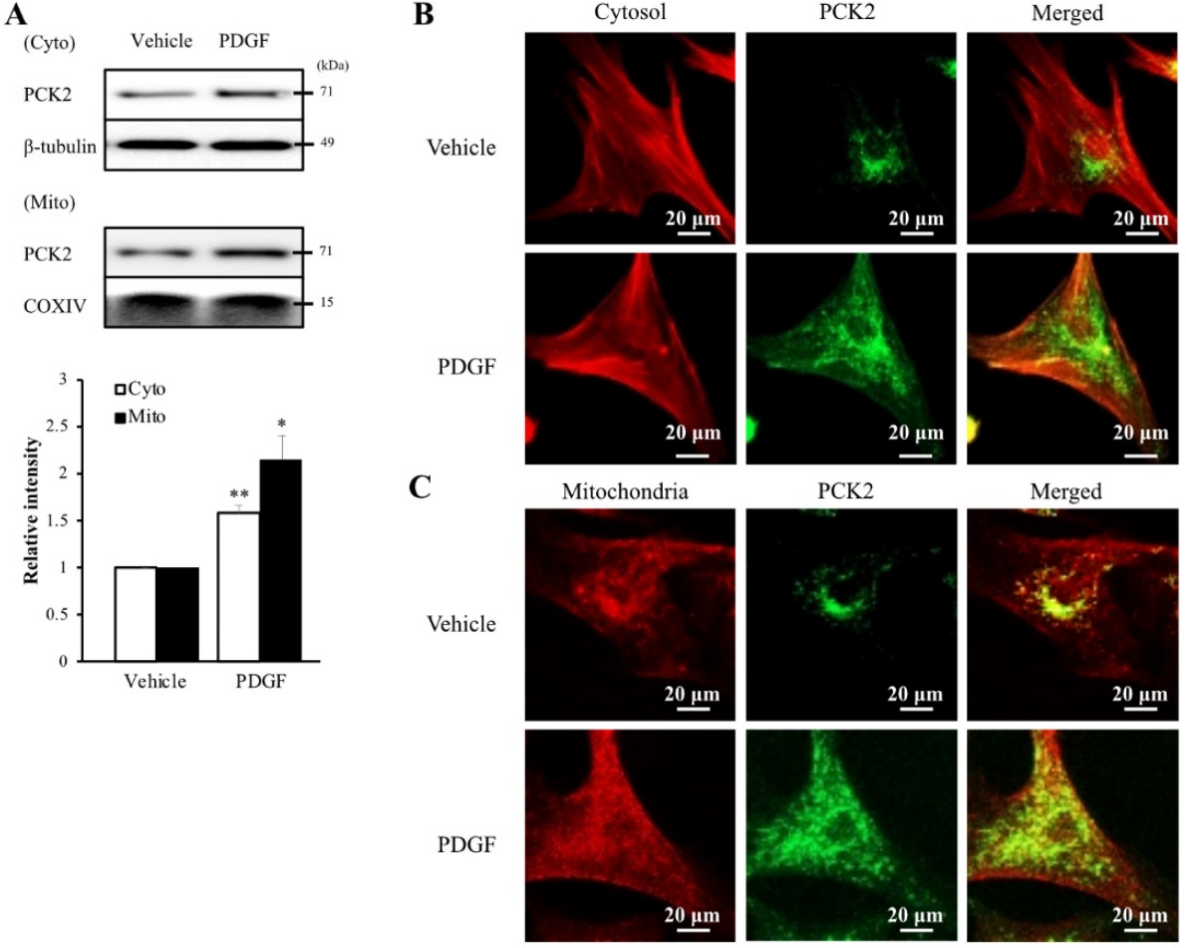
** Intracellular localization of PCK2 on PDGF stimulation. (A)** Protein level of PCK2 in cytosol and mitochondria of hVSMCs. PCK2 in the cytosolic fraction (cyto) was analyzed by western blot analysis. β-tubulin was used as an internal control for cytosolic protein. PCK2 in the mitochondrial fraction (mito) was analyzed by western blot analysis. COXIV protein served as an internal control for mitochondrial protein. Quantitative analysis of PCK2 protein expression in cytosolic and mitochondrial fractions. Experiments shown are representative of three independent experiments. **(B and C)** PCK2 localizes to the cytosol and mitochondria in PDGF-stimulated hVSMCs. Intracellular localization of PCK2 via immunocytochemistry. Cells were treated with 10 ng/mL PDGF for 24 h, and stained. Cytosol was detected with anti-β-tubulin (red) and PCK2 (green) antibodies. Mitochondria were visualized using MitoTracker (red) and PCK2 (green) staining. A digitally merged image of cytosol and mitochondria signals reveals colocalization of PCK2. Images are representative of four independent experiments.

To investigate the PI3K-Akt pathway of PDGF-induced PCK2 expression, hVSMCs were pretreated with pharmacological inhibitors of PI3K/Akt pathways, including PI3K (LY294002, LY) and Akt (Akt inhibitor IV, AI) inhibitors for 1 h and stimulated with platelet-derived growth factor (PDGF) (10 ng/ml) for 24 h. The protein and mRNA levels of PCK2 in PDGF-treated cells were inhibited by LY and AI (Figure 3A). Moreover, PDGF-induced PCK2 expression was inhibited in VSMCs transfected with siRNA against *Akt2* and *Akt3,* but not in those transfected with siRNA against* Akt1* (Figure 3B‒D). We also investigated PDGF-induced FoxO phosphorylation in the PCK2-associated signaling pathway. Likewise, VSMCs were pretreated with LY and AI for 1 h and stimulated with PDGF (10 ng/ml) for 24 h. Phosphorylation of FoxO1 and FoxO3 in PDGF-treated cells was inhibited by LY and AI, but not by FoxO4 (Figure 3E). VSMCs were transfected with siRNA against *Akt2* and *Akt3* for 48 h and stimulated with PDGF for 24 h. Phosphorylation of FoxO1 and FoxO3, increased by PDGF, was considerably inhibited in *Akt2*- and *Akt3*-depleted cells (Figure 3F and G). We also transfected VSMCs with siRNA against FoxO1 and FoxO3 for 48 h and stimulated them with PDGF for 24 h. PCK2 expression, increased by PDGF, was substantially inhibited *FoxO1*- and *FoxO3*-depleted cells (Figure 3H and I). These results suggested that PDGF increased PCK2 expression via PI3K/Akt and FoxO signaling pathways, particularly via Akt2, Akt3, FoxO1, and FoxO3. To assess subcellular localization of PCK2, we extracted cytosolic and mitochondrial proteins from hVSMCs treated with PDGF (Figure 4A) and performed immunofluroscence analysis (Figure 4B). Interestingly, PCK2 expression increased in both cytosol and mitochondria in hVSMCs treated with PDGF.

### Attenuation of VSMC proliferation in wire-injured vasculature of *Pck2* knock-out mouse

**Figure 5:**
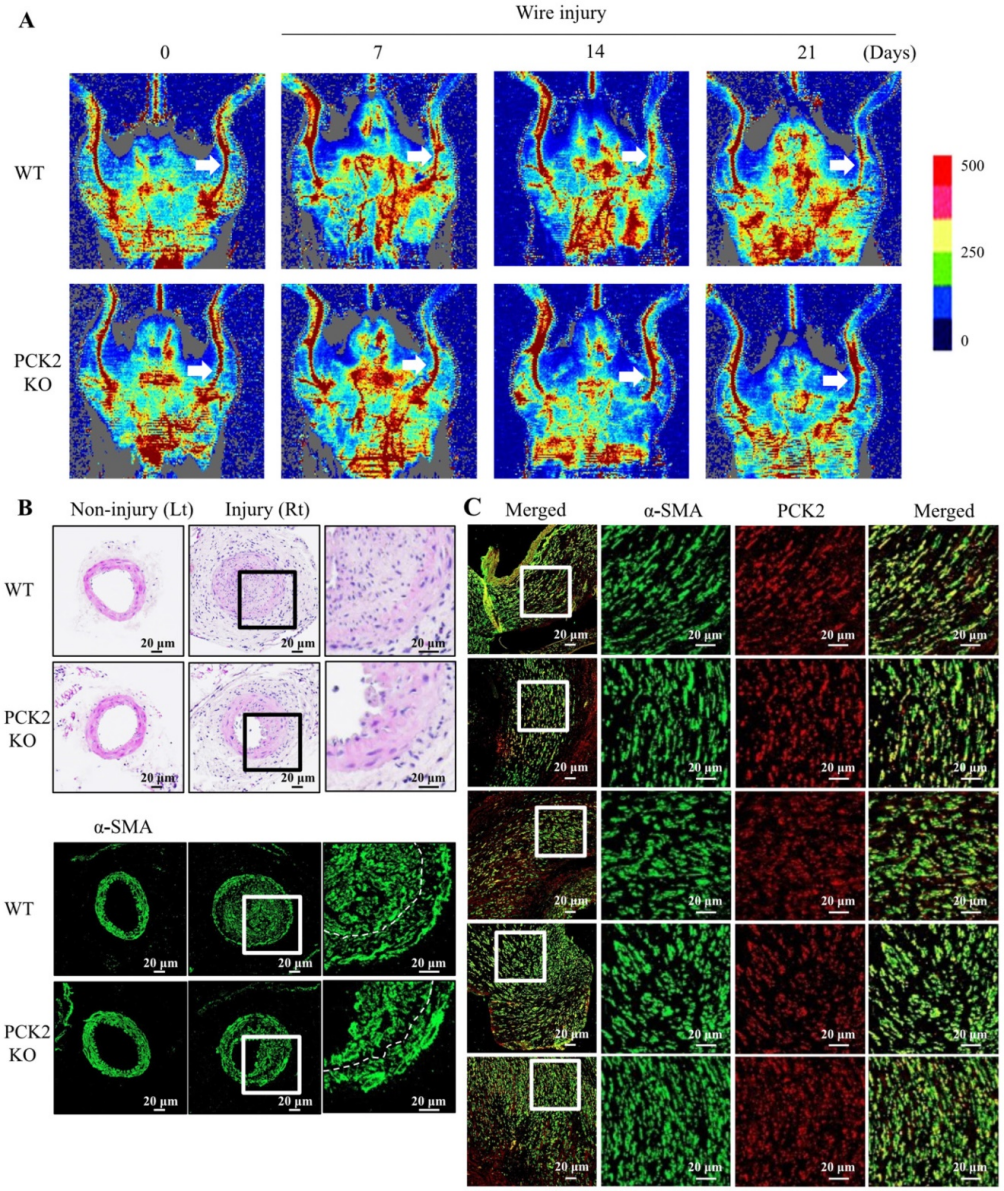
** Identification of pivotal role that PCK2 plays in vascular restenosis and atherosclerosis. (A)** Time courses of changes in blood flow in wire-injured femoral arteries of WT and *Pck2* KO mice. Blood flow in the femoral arteries of WT and *Pck2* KO mice at 0 (before wire-injury), 1, 2, and 3 weeks after injury was monitored using a laser Doppler perfusion imaging (LDPI). Arrows indicate blood flow in injured femoral arteries. Photographs are representative of five independent experiments. **(B)** Cross sections of mouse femoral arteries were prepared at four weeks after wire injury and stained with H&E. Vascular smooth muscle cells were stained with anti-α-SMA antibody. Dashed line defines media and neointima layer. Images are representative of six independent experiments. **(C)** Human femoral atheroma retrieved when performing endarterectomy of the common femoral artery were stained with anti-α-SMA and PCK2 antibodies. Images are representative of five independent experiments.

We investigated the role of PCK2 in the formation of neointimal hyperplasia *in vivo*. Blood flow in wire-injured femoral arteries was higher in *Pck2* knockout mice than that in WT mice 3 weeks post-injury (Figure 5A). Doppler images of blood flow over time (before wire-injury, and 7, 14, and 21 days after wire-injury) are shown. H&E staining and immunofluorescence analysis revealed that *Pck2* knockout remarkably reduced neointimal hyperplasia (Figure 5B).

### PCK2 expression in VSMCs of human femoral atheroma

To examine the expression of PCK2 in human femoral atheroma, we collected five femoral atheroma samples during endarterectomy of the common femoral artery. The proliferation of VSMCs and colocalization of PCK2 with α-SMA in all femoral atheroma samples was observed (Figure 5C).

## Discussion

We identified six DEGs from the four gene expression datasets. Of the six DEGs, only *PCK2* and *RGS1* displayed identical expression patterns in wire-injured femoral arteries of mice and the gene expression datasets. Moreover, PDGF-induced VSMC proliferation was attenuated in hVSMCs transfected with *PCK2* siRNA, but not in cells transfected with *RGS1* siRNA. Notably, RNA sequencing of hVSMCs treated with *PCK2* siRNA revealed that PCK2 was associated with both the PI3K-Akt and FoxO signaling pathways. We also demonstrated that Akt2, Akt3, FoxO1, and FoxO3 were associated with PDGF-induced PCK2 expression in hVSMCs. In addition, PCK2 had a direct effect on VSMC proliferation when it was inhibited with an inhibitor. Remarkably, wire injuries in *Pck2*-KO mice had decreased neointimal hyperplasia and increased blood flow to the injured extremity as observed by Doppler imaging. Lastly, we also demonstrated that PCK2 was expressed in proliferating VSMCs in human femoral atherosclerotic plaques.

High-throughput technologies have been indispensable tools for investigating candidate biomarkers and biological pathways 23. Large-scale investigations of transcriptional changes related with biological situations of interest have made it possible to identify differentially expressed genes associated with diseases 24, 25. However, differences in laboratory protocols, analytical methods, and small sample sizes have led to low reproducibility and consistency between studies. Independent studies have also attributed this discrepancy to the heterogeneity present in many disease subtypes 26. Therefore, to overcome these limitations, we identified common DEGs from different datasets. Of these, two datasets included intact and atherosclerotic carotid arteries (or plaques), whereas others included early or stable atherosclerotic plaques and advanced or unstable atherosclerotic plaques in the carotid arteries. Using this strategy, we narrowed down candidate genes involved in disease exacerbation (i.e., advanced carotid plaque lesions and plaque rupture) by selecting genes that were differentially expressed between normal and disease samples. Thereafter, we investigated which candidate genes were involved in VSMC proliferation that had not been studied by the authors of the datasets that were analyzed in this study.

We demonstrated that the Akt-FoxO-PCK2 pathway was associated with PDGF-induced VSMC proliferation, particularly via Akt2, Akt3, FoxO1, and FoxO3. The PI3K/Akt/FoxO signaling pathway is crucial for multiple cellular functions, including cell proliferation and survival 27. Reportedly, knockdown of *Akt1* on an apolipoprotein E knockout background (ApoE^-/-^ Akt1^-/-^) increased the levels of inflammatory mediators and reduced eNOS phosphorylation, leading to severe atherosclerosis and occlusive coronary artery 28. Deletion of *Akt1* also reduced VSMC proliferation and migration, thereby inducing plaque vulnerability 29. Rensing et al. discovered that *ApoE^-/-^ Akt2^-/-^* double knockout mice exhibited smaller but more complicated carotid atherosclerotic plaques 30. Additionally, they demonstrated that *Akt2* knockout impaired collagen synthesis, VSMC proliferation, and migration and altered the expression of metalloproteinases and tissue inhibitors of metalloproteinases. Although it is mainly expressed in the brain, *Akt3* also has a protective function in atherosclerosis. Ding et al. demonstrated that genetic ablation of *Akt3* in ApoE^-/-^ mice led to a two-fold increase in atherosclerotic lesions and that Akt3 suppressed foam cell formation by specifically inhibiting the accumulation of macrophage cholesteryl esters 31.

FoxOs induce the expression of pro-apoptotic members of the Bcl2-family and inhibit cell growth and apoptosis. Therefore, FoxOs have been studied as a key class of tumor suppressors in a range of malignancies 32. Notably, Akt directly inhibits FoxOs by phosphorylation and regulates cell survival, growth, and proliferation. Previous studies have shown that Akt1 plays a protective role against atherosclerosis in VSMCs and that FoxO3a activity is elevated in VSMCs atherosclerotic plaques in human 29, 33. *In vivo* experiments with transgenic mice expressing Akt1 specifically activated in arterial VSMCS demonstrated that Akt1 inhibited apoptosis in VSMCs. Moreover, it inhibited negative remodeling after carotid ligation through FoxO3a and Apaf1 34. Yu et al. demonstrated that FoxO3a activation promoted the expression of MMP13 and induced apoptosis in VSMCs and breakdown of the extracellular matrix 35. Additionally, Deng et al. reported that Akt up-regulates a key osteogenic transcription factor, runt-related transcription factor 2, and induces VSMC calcification via FoxO1/3 36. In our study, both Akt2 and Akt3 increased the phosphorylation of FoxO1 and FoxO3. In addition, we demonstrated that knockdown of FoxO1 and FoxO3 in VSMCs decreased the expression of PCK2. Therefore, our results are consistent with those of previous studies, i.e., the proapoptotic function of FoxO1/3 in VSMCs is inhibited by Akt2/3. Moreover, we elucidated that PCK2 is a downstream component of the Akt-FoxO pathway that regulates VSMC proliferation. Given that Akt1 is expressed predominantly in VSMCs 27 and the roles of Akt2 and Akt3 in VSMC proliferation have not yet been thoroughly studied 37, this study is distinctive in that it investigates the effects of Akt2/3-FoxO1/3-PCK2 pathway on VSMC proliferation.

Phosphoenolpyruvate carboxykinase (PEPCK) has a key role in gluconeogenesis. Although the role of PEPCK-C (cytoplasmic form; PCK1) has been thoroughly investigated in diabetes and obesity 38, that of its counterpart isozyme, PEPCK-M (mitochondrial form, PCK2), remains unexplored. Recent reports have revealed that a potential role of PCK2 is to promote tumor growth and cell proliferation. These studies indicated that glucose restriction enhanced phosphoenolpyruvate synthesis from glutamine via PCK2 39, 40. Moreover, PCK2 expression was necessary for the maintenance of tumor cell proliferation *in vitro* under glucose-limiting circumstances and for tumor growth *in vivo*.^41^. Ma et al. reported that RNA-binding protein Lin28a regulates cardiomyocyte hypertrophic growth by increasing PCK2 transcripts and facilitating metabolic repatterning in response to cardiac stress 42. VSMCs are dependent on glycolysis rather than mitochondrial glucose oxidation 43. When switched from the contractile phenotype to the synthetic phenotype of VSMCs in atherosclerotic plaques, their dependency on glycolysis increases with a decrease in glucose oxidation 44. It was reported that the expression of hexokinase 2, an enzyme that catalyzes phosphorylation of glucose to glucose-6-phoshate, led to a three-fold increase in the human carotid atherosclerotic plaque cap, which may contribute to increased glycolytic activity and decreased oxidative phosphorylation in VSMCs 45. To date, little attention has been paid to PCK2's role in metabolic adaptation on VSMC proliferation.

Wire injury in an animal model reproduces atherosclerotic conditions, such as neointimal formation post-stent implantation, balloon angioplasty, or endarterectomy, owing to limitations in conducting clinical studies 46. In our *in vivo* study, intimal hyperplasia in wire-injured femoral artery was substantially attenuated in *Pck2*-deficient mice compared with that in WT control mice. Doppler imaging indicated that a wire-injured *Pck2* KO mouse exhibited improved arterial perfusion in the injured extremity compared with wire-injured wild-type mice. Moreover, we demonstrated that PCK2 levels were measurable in human atherosclerotic plaques of the femoral arteries. Interestingly, VSMCs in human atherosclerotic plaques had the most expression of PCK2, suggesting that PCK2 can be utilized as a therapeutic target for preventing restenosis after interventional treatment of peripheral atherosclerotic lesions. This study has some limitations. First, in silico analysis, the DEGs were derived from datasets of atherosclerotic lesions. We validated their expression in wire-injured femoral arteries in mice. It is possible that the DEGs other than *PCK2* and *RGS1* were not validated because changes following wire-injury are acute phase and atherosclerotic lesions are chronic. Second, we did not elaborate on how PCK2 affects VSMC proliferation, specifically as a metabolic adaptation. It should be the goal of our future studies.

## Conclusion

Although the use of stents has improved the patency rates in femoropopliteal disease, nearly 30-40% of patients experience in-stent restenosis within 2 years of implantation 47. To reduce neointimal hyperplasia, local delivery of drugs via drug-coated balloons or drug-coated stents has been a widely used approach. In the clinical scenario, drug-eluting balloon angioplasty presents a superior outcome to uncoated balloon angioplasty for the treatment of in-stent restenosis of the femoropopliteal arteries 48. However, it is necessary to develop new therapeutic targets to prevent restenosis and to ensure an optimal clinical outcome. In this study, we present evidence that PCK2 is primarily expressed by VSMCs and plays a protective role in atherosclerotic injury. Pharmacodynamic techniques are evolving to activate specific downstream signaling pathways to optimize treatment efficacy and reduce side effects, such as immune-mediated disorders 49. Therefore, our results suggest that targeting PCK2, a downstream component of the Akt-FoxO-PCK2 pathway, is a potential valuable strategy for the treatment of atherosclerotic lesions associated with vascular injury.

## Supplementary Material

Supplementary figures.

Supplementary tables.
