# Supplementary material for: Role of PCK2 in the proliferation of vascular smooth muscle cells in neointimal hyperplasia
Source: Int J Biol Sci. 2022 Aug 8;18(13):5154–67. doi: 10.7150/ijbs.75577 (PMC9379418; doi:10.7150/ijbs.75577)
Supplement: Supplementary file 1 — Supplementary figures. [file ijbsv18p5154s1.pdf]

Supplementary Figures

Figure S1

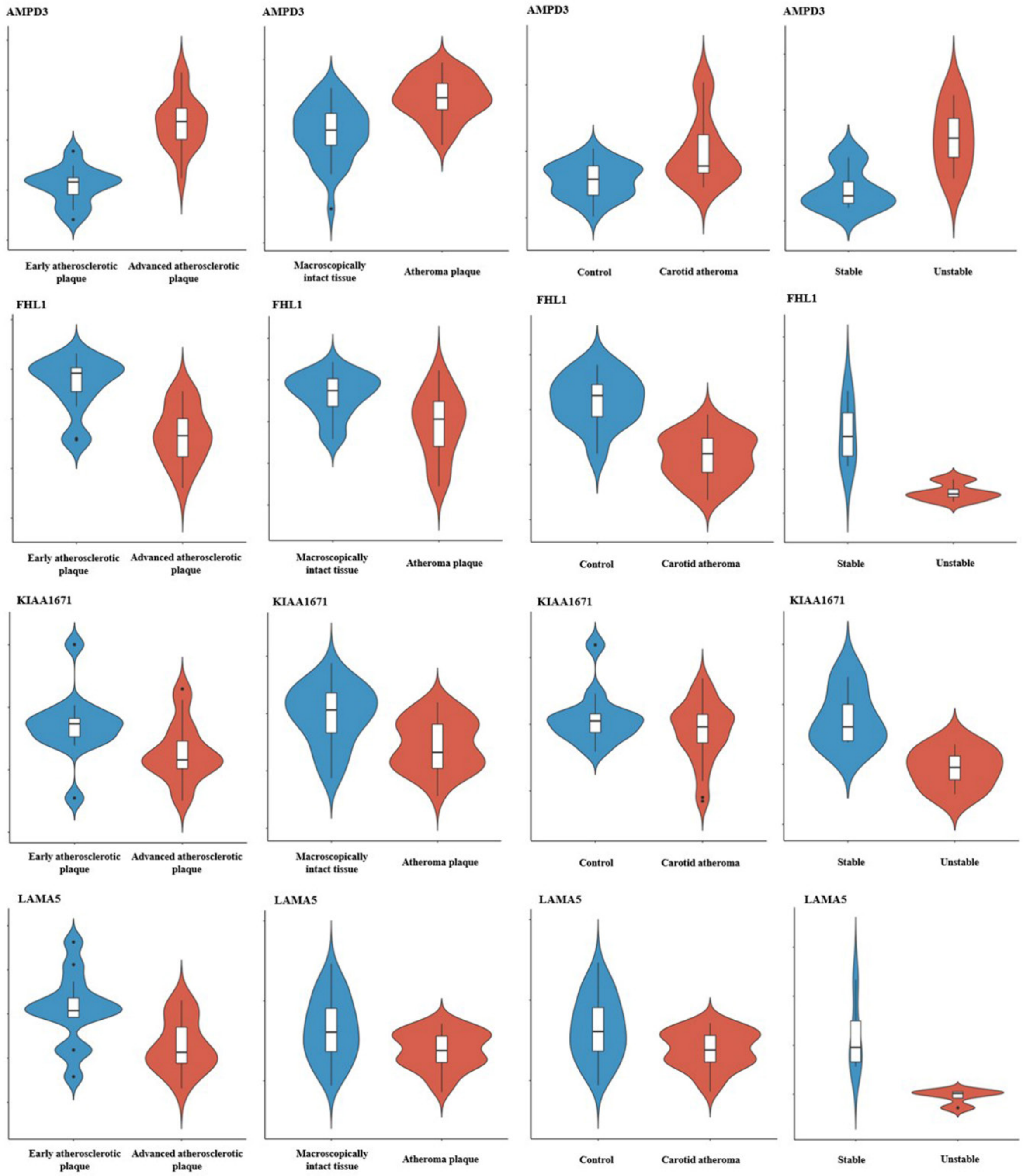

Figure S1. Violin plots of four unvalidated differentially expressed genes in wire-injured femoral arteries. The violin plots are shown for each gene and from the left are GSE28829, GSE43292, GSE100927, and GSE120521.

Figure S2

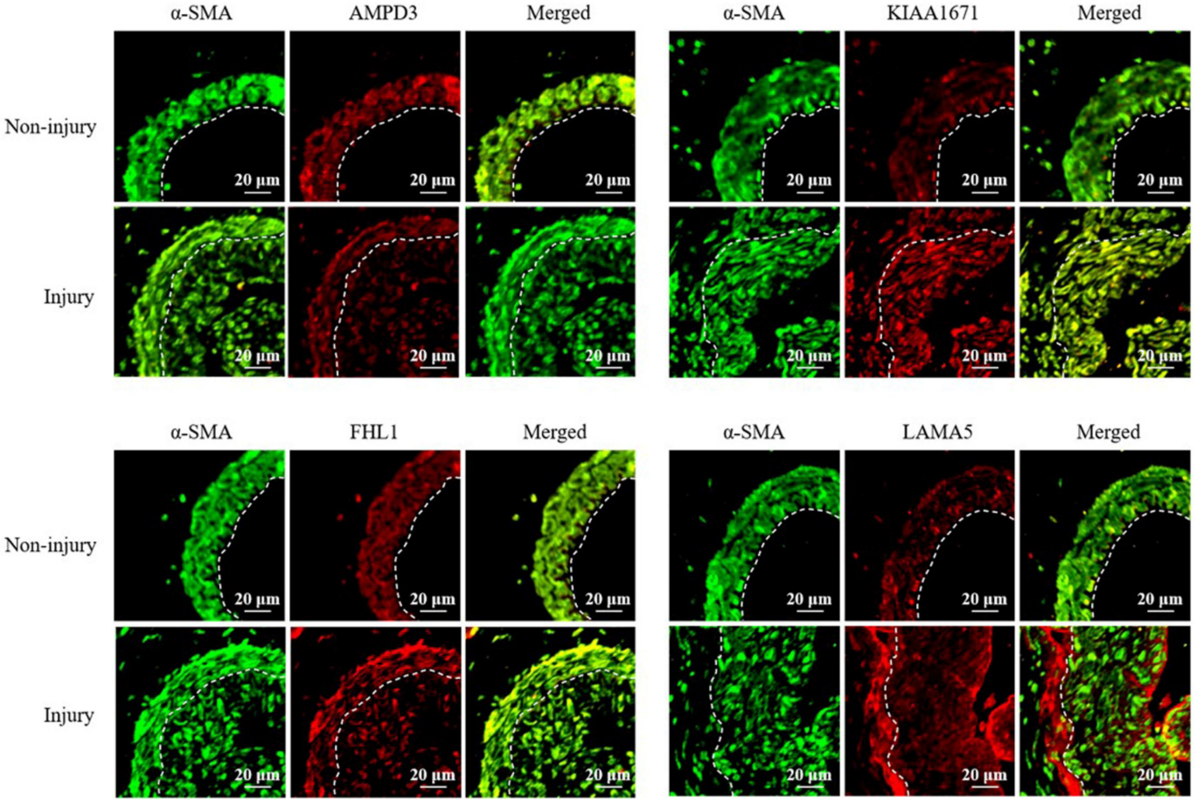

Figure S2. Involvement of AMPD3, KIAA1671, FHL1, and LAMA5 in wire-injured femoral arteries. AMPD3, KIAA1671, FHL1, and LAMA5 in the indicated media and neointima were stained with anti-AMPD3, anti-KIAA1671, anti-FHL1, and anti-LAMA5 antibodies, respectively. The gene expression levels of the non-injured and injured media were compared. Vascular smooth muscle cells were stained with anti-α-SMA antibody. Images are representative of 3–5 independent experiments.

Figure S3

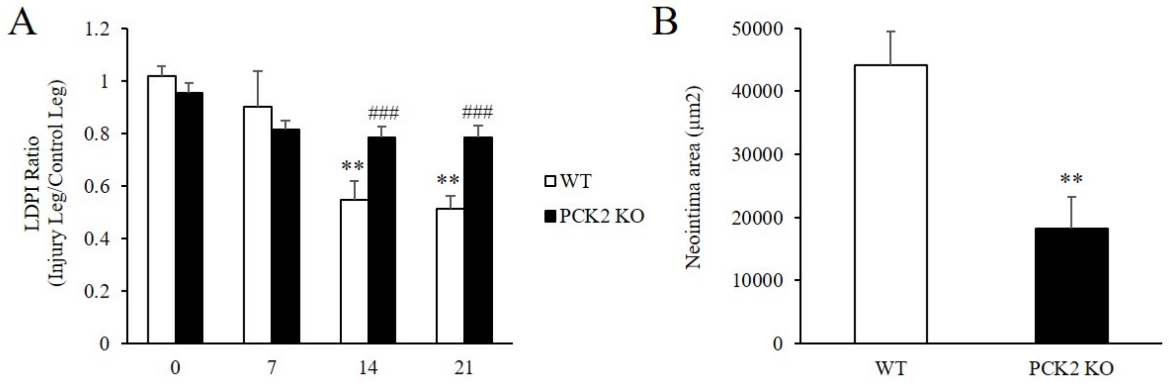

Figure S3. Quantification of blood flow and neointimal volume in wire-injured femoral arteries in WT and PCK2 KO mice. (A) Blood flow was measured at 0 (before surgery), 1, 2, 3 and 4 weeks after wire-injury to the femoral artery and quantified via the ratio of LDPI color pixels. Datas are representative of 5 independent experiments. **P < 0.01 vs. corresponding value in 0 (before surgery), ###P < 0.001 vs. corresponding value in WT mice. (B) The neointimal volume of the injured femoral artery section was measured using an image analyzer. Images are representative of 6 independent experiments. **P < 0.01 vs. WT mice.
